# Novel insights of ferroptosis in atherosclerosis progression

**DOI:** 10.3389/fcell.2026.1851553

**Published:** 2026-05-29

**Authors:** Pifeng Liang, Yuxiang Huang

**Affiliations:** 1 Department of Nursing, The People’s Hospital of Longhua Shenzhen, Shenzhen, China; 2 Department of Cardiology, The People’s Hospital of Longhua Shenzhen, Shenzhen, China

**Keywords:** atherosclerosis, endothelial dysfunction, ferroptosis, inflammation, iron homeostasis, macrophage polarization, oxidative stress

## Abstract

Atherosclerosis (AS) is the principal pathological basis of multiple cardiovascular and cerebrovascular diseases and remains a major cause of morbidity and mortality worldwide. Accumulating evidence indicates that ferroptosis, a form of iron-dependent regulated cell death driven by lipid peroxidation, is intricately involved in the initiation and progression of AS. Aberrant iron accumulation, redox imbalance, and impaired antioxidant defenses contribute to endothelial dysfunction, macrophage activation, vascular smooth muscle cell phenotypic switching, lipid metabolic disturbance, and amplified immune-inflammatory responses, thereby accelerating plaque formation and destabilization. Key regulatory systems, including iron transport pathways, the GSH/GPX4 axis, FSP1-CoQ10 signaling, and mitochondrial iron metabolism, have emerged as critical determinants of ferroptotic susceptibility in vascular cells. Experimental studies further suggest that ferroptosis inhibition can attenuate oxidative injury, reduce inflammatory mediator release, improve lipid metabolic profiles, and stabilize atherosclerotic plaques. This review summarizes the mechanisms governing cellular iron homeostasis and ferroptosis, discusses the multifaceted roles of ferroptosis in the major cellular and molecular events underlying AS progression, and highlights the therapeutic promise of targeting ferroptotic pathways in atherosclerotic disease.

## 1 Introduction

Atherosclerosis (AS) is a chronic inflammatory vascular disease and the pathological foundation of coronary artery disease, ischemic stroke, and other major cardiovascular disorders (Russo et al., 2025; Chen W. et al., 2022). Its development is driven by metabolic and hemodynamic risk factors, including hyperlipidemia, hypertension, diabetes mellitus, obesity, and smoking (Kong et al., 2022; Jebari-Benslaiman et al., 2022). At the cellular level, AS begins with endothelial dysfunction, lipid retention, and monocyte recruitment into the arterial intima, followed by macrophage foam-cell formation, vascular smooth muscle cell remodeling, extracellular matrix deposition, calcification, and necrotic core expansion (Jiao et al., 2025; Zhang et al., 2025). These interconnected processes progressively promote plaque growth and destabilization, ultimately leading to thrombosis and life-threatening cardiovascular events.

Ferroptosis, an iron-dependent form of regulated cell death characterized by excessive lipid peroxidation and impaired antioxidant defense, has emerged as a critical mechanism linking iron overload, oxidative stress, lipid metabolic disorder, and vascular inflammation in AS (Zhang M. et al., 2024; Li C. et al., 2024; Elkammash et al., 2024). Key ferroptosis-related pathways, including the GSH/GPX4 axis, FSP1–CoQ10 system, ACSL4-mediated lipid peroxidation, mitochondrial iron metabolism, and hepcidin–ferroportin signaling, may regulate endothelial injury, macrophage polarization, foam-cell formation, vascular smooth muscle cell phenotypic switching, and plaque instability (Zhao et al., 2025; Jiang et al., 2025; Wan et al., 2023; Malhotra et al., 2019). Therefore, this review summarizes the molecular regulation of ferroptosis and discusses its pathogenic and therapeutic relevance in AS progression.

## 2 Mechanisms regulating cellular iron homeostasis

### 2.1 Cellular iron metabolism

In the circulation, Fe^3+^ is delivered into cells primarily through transferrin (Tf) binding and subsequent engagement of transferrin receptor 1 (TfR1) (Guo Q. et al., 2025; Yesiltepe et al., 2026). Under conditions of iron overload, however, iron may also enter cells as non-transferrin-bound iron (NTBI), a form not complexed with Tf or other iron-binding proteins (Arezes et al., 2013; Pinto et al., 2014). The accumulation of labile Fe^2+^ is a central driver of the Fenton reaction and thus the principal basis of iron-mediated cytotoxicity (Kafina and Paw, 2017; Cain and Smith, 2021). Notably, the Tf/TfR1 axis is a key determinant of ferroptotic susceptibility because it governs the intracellular labile iron pool (LIP). TfR1 upregulation, commonly observed under inflammatory or oxidative stress, enhances iron uptake and Fe^2+^ accumulation, thereby accelerating Fenton chemistry and lipid peroxide formation (Yu et al., 2021; Wu et al., 2025; Viaud et al., 2020; Ru et al., 2024). As a result, vascular cells, including endothelial cells and macrophages, become more vulnerable to ferroptosis. Inflammatory pathways such as TLR4/NF-κB and HIF-1α can transcriptionally induce TfR1, linking immune activation to ferroptotic sensitivity (Wang et al., 2025; Chen et al., 2026; Yang J. et al., 2026). Ferritin and ferroportin (FPN) are major regulators that restrain intracellular iron accumulation and limit toxicity. FPN is internalized and degraded by hepcidin, promoting iron retention, whereas nuclear receptor coactivator 4 (NCOA4) mediates ferritinophagy and thereby increases intracellular iron availability (De Domenico et al., 2009; Santana-Codina and Mancias, 2018). Ferritin serves as an essential buffering system by sequestering excess iron in a redox-inactive form; however, NCOA4-dependent ferritinophagy releases stored iron, markedly expands the LIP, and amplifies ferroptosis through ROS-driven lipid peroxidation (Santana-Codina and Mancias, 2018; Ning et al., 2026). Although ferritin upregulation may transiently confer protection, persistent inflammatory stress often overrides this effect (Kotla et al., 2022). The hepcidin–FPN axis further regulates ferroptotic sensitivity. During inflammation, IL-6/STAT3 signaling strongly induces hepcidin expression, promoting FPN degradation and intracellular iron sequestration (Camaschella et al., 2020; Wrighting and Andrews, 2006; Li Z. D. et al., 2022). This iron-retentive state intensifies oxidative stress, lipid peroxidation, and ultimately ferroptotic cell death. In atherosclerotic lesions, elevated hepcidin has been associated with macrophage iron loading, pro-inflammatory polarization, and increased ferroptotic susceptibility (Wunderer et al., 2020; Sullivan, 2007).

### 2.2 Lipid and amino acid-driven ferroptosis

Iron deposition-driven lipid peroxide accumulation constitutes a pivotal initiating event in ferroptosis. Polyunsaturated fatty acids (PUFAs), which are highly enriched in cellular membranes, are particularly susceptible to reactive oxygen species (ROS)-mediated oxidation owing to the presence of multiple carbon–carbon double bonds, thereby serving as a major substrate source for lipid peroxidation (Kim et al., 2023; Mortensen et al., 2023). PUFA peroxidation proceeds through two principal mechanisms: a non-enzymatic route driven by Fenton chemistry and a tightly regulated enzymatic route mediated predominantly by lipoxygenases (LOXs) (Gaschler and Stockwell, 2017; Zhang X. et al., 2022). Within this framework, acyl-CoA synthetase long-chain family member 4 (ACSL4) and lysophosphatidylcholine acyltransferase 3 (LPCAT3) function as crucial determinants of ferroptotic sensitivity by facilitating PUFA activation, membrane incorporation, and subsequent peroxidation (Cui and Ye, 2026; Sun et al., 2025). Consistent with this role, inhibition of ACSL4 or LPCAT3 has shown substantial efficacy in attenuating ferroptotic cell death. Both enzymatic and non-enzymatic lipid oxidation pathways converge on the generation of lipid peroxides, together with highly reactive secondary products, including malondialdehyde (MDA) and 4-hydroxynonenal (4-HNE) (Xu et al., 2020; Zhang X. et al., 2023). These toxic aldehydic by-products profoundly perturb membrane biophysical properties and structural integrity, promote membrane permeabilization, and ultimately culminate in catastrophic membrane rupture and cell death (Haro et al., 2023). Counterbalancing this lipid peroxidation cascade, the glutathione (GSH)/GPX4 axis represents the canonical cellular defence system against ferroptosis (Duo et al., 2025; Lee et al., 2021). GSH biosynthesis critically depends on glutamine and cysteine metabolism, amino acid availability is intimately coupled to ferroptosis susceptibility (Jiang Y. et al., 2024; Liu et al., 2020). In parallel, the γ-glutamyl cycle and the GCH1–BH4 pathway constitute complementary antioxidant systems that can restrain ferroptotic damage independently of the canonical GSH/GPX4 machinery (Zhang R. et al., 2022; Chen Y. et al., 2022). In addition, ferroptosis suppressor protein 1 (FSP1) acts as a potent parallel safeguard against ferroptosis by catalysing the NAD(P)H-dependent regeneration of reduced coenzyme Q10, thereby intercepting lipid radical propagation and reinforcing membrane-associated antioxidant capacity (Hadian, 2020; Silver et al., 2026).

### 2.3 Mitochondrial ferroptosis

Mitochondria serve as major hubs for iron utilization and accumulation and are also key organelles involved in ferroptotic injury (Zhao et al., 2024). Mitochondrial iron trafficking across the inner membrane is mediated by mitoferrin 1/2 (Mfrn1/2) and by the Fe-S cluster-binding protein CDGSH iron sulfur domain 1 (CISD1) (Richardson et al., 2010). Mitochondrial ferritin (FtMt), analogous to cytosolic ferritin, oxidizes Fe^2+^ into the less redox-active Fe^3+^ form (Liu Y. et al., 2023). Mitochondrial iron overload can likewise initiate Fenton chemistry and promote membrane damage (Ciambellotti et al., 2021; Bradley et al., 2025). Moreover, superoxide anions generated by electron leakage from mitochondrial electron transport chain (ETC.) complexes I and III can be converted by superoxide dismutase (SOD), and the resulting oxidants subsequently react with Fe^2+^-containing PUFA substrates and oxygen to generate lipid peroxides, thereby driving ferroptotic cell death (Mortensen et al., 2023; Andrés et al., 2023). Dihydroorotate dehydrogenase (DHODH), located on the inner mitochondrial membrane, functions as a coenzyme Q10 reductase analogous to FSP1 and can likewise promote the generation of reduced coenzyme Q10, thereby exerting a GPX4-like protective effect (Mao et al., 2021; Cao J. et al., 2025). Notably, defective or excessive mitophagy may conversely amplify ferroptotic and inflammatory injury during AS progression (Zhang Y. et al., 2023; Xu S. et al., 2024). When mitophagic clearance is insufficient, damaged mitochondria accumulate and continuously release ROS, mitochondrial DNA, cardiolipin oxidation products, and other danger-associated molecular patterns (Wang et al., 2023; Deus et al., 2020). These signals can activate TLR9, cGAS-STING, NF-κB, and the NLRP3 inflammasome, thereby promoting the secretion of IL-1β, IL-18, IL-6, and TNF-α (Tao et al., 2024; Liu et al., 2021; Luan et al., 2023). In macrophages, this inflammatory milieu may reinforce M1-like polarization, impair cholesterol efflux, accelerate foam-cell formation, and contribute to necrotic core expansion (Blagov et al., 2023). In endothelial cells, mitochondrial ROS and lipid peroxidation can reduce nitric oxide bioavailability, increase VCAM-1 and ICAM-1 expression, and enhance monocyte adhesion (Wang and He, 2020). Besides, mitophagy–ferroptosis imbalance may promote phenotypic switching, senescence-associated inflammatory mediator release, and plaque instability (Zhang S. et al., 2022; Lin et al., 2021). Impaired mitophagy may also enhance ACSL4-dependent PUFA-phospholipid peroxidation, NCOA4-related iron mobilization, and GPX4 depletion, thereby sensitizing vascular cells to ferroptosis (Li K. et al., 2024; Zhu et al., 2025). Thus, mitophagy represents a critical regulatory node linking mitochondrial iron metabolism, ferroptosis, oxidative stress, and vascular inflammation in AS (Zhang Y. et al., 2023). Targeting mitophagy–ferroptosis crosstalk may therefore provide a promising therapeutic strategy for limiting endothelial dysfunction, macrophage-driven inflammation, and plaque destabilization.

## 3 Ferroptosis drives the progression of atherosclerosis

### 3.1 Ferroptosis promotes endothelial dysfunction

Endothelial cells (ECs) orchestrate vascular homeostasis through paracrine and endocrine signaling to vascular smooth muscle cells, platelets, and leukocytes. By secreting vasoactive mediators, including nitric oxide (NO), prostacyclin (PGI_2_), endothelin-1 (ET-1), angiotensin II (Ang II), VCAM-1, and PDGF (Wang and He, 2020; Galley and Webster, 2004; Celermajer, 1997; Krüger-Genge et al., 2019). ECs dynamically regulate vasomotor tone, smooth muscle proliferation, and leukocyte adhesion (Reriani et al., 2010). Concurrently, EC-derived anticoagulant, antiplatelet, and fibrinolytic factors preserve hemostatic equilibrium. Disruption of endothelial integrity precipitates dysregulated vascular tone, prothrombotic shifts, and enhanced monocyte recruitment, thereby initiating atherogenesis (De Pablo-Moreno et al., 2022; Palomo et al., 2008). Emerging evidence identifies ferroptosis as a pivotal driver of endothelial dysfunction in atherosclerosis. Chronic iron overload exacerbates EC injury by depleting antioxidant defenses (SOD, CAT, eNOS), upregulating COX-2, and altering prostanoid release, collectively accelerating plaque development (Marques et al., 2019). Conversely, pharmacological suppression of ferroptosis with ferrostatin-1 attenuates aortic EC death, limits lipid peroxidation and intracellular iron accumulation, and restores the SLC7A11–GPX4 axis. Similarly, ACSL4 inhibition by berberine suppresses lipid deposition and plaque progression *in vivo* and *in vitro* (Bai et al., 2020; Hong et al., 2024). Oxidized LDL further promotes EC ferroptosis via GPX4 downregulation, amplifying inflammation and monocyte adhesion—effects readily reversed by ferroptosis inhibitors (Yang et al., 2020). Moreover, HO-1 upregulation alleviates intracellular iron overload and ox-LDL-induced ferroptosis, partially rescuing endothelial viability (Guo W. et al., 2025).

### 3.2 Macrophage ferroptosis

Macrophages constitute the predominant immune population within AS plaques and orchestrate disease progression through phenotypic plasticity, classically dichotomized into pro-inflammatory M1 and reparative M2 subsets (Eshghjoo et al., 2022). In AS, macrophages are derived primarily from circulating monocytes. These cells can exert both pro-inflammatory and anti-inflammatory effects, a functional duality that is closely linked to their polarization state, most commonly categorized as M1 and M2 phenotypes (Amengual and Barrett, 2019; Hou et al., 2023). As a major macrophage subtype in advanced AS lesions, M1 macrophages promote inflammation through the release of cytokines such as TNF-α, IL-1β, and IL-6 (Wu et al., 2023; Deng et al., 2025). In addition, M1-derived matrix metalloproteinases, including MMP-1, MMP-3, and MMP-9, degrade collagen fibers within the fibrous cap, a process widely considered to contribute to the instability of vulnerable plaques (Di Nubila et al., 2024; Newby, 2007). In contrast, M2 macrophages activate anti-inflammatory regulatory programs through secretion of IL-10, TNF-β, and chemokines such as CCL17, CCL22, and CCL24, thereby enhancing plaque stability (Zhang L. et al., 2024). Previous studies have shown that Fe^2+^ accumulates selectively in inflammation-activated macrophages (Ma et al., 2023). Excessive iron deposition within the arterial wall promotes lipid accumulation, endothelial activation, and reduced bioavailability of NO; moreover, iron overload also stimulates monocyte recruitment during this process. High levels of labile iron further increase arginase activity, suppress nitric oxide synthase (NOS) activity, weaken the antioxidant capacity of macrophages, and promote the secretion of MMPs, thereby facilitating extracellular matrix degradation and plaque rupture (Cornelissen et al., 2019; Vinchi et al., 2014). Conversely, the specific ferroptosis inhibitor ferrostatin-1 (Fer-1) can attenuate ox-LDL-induced reductions in macrophage viability and lipid peroxidation, thereby delaying AS progression (You et al., 2023; Li et al., 2023).

The interplay between ferroptosis and macrophage polarization in AS is governed by converging redox and metabolic signaling networks. Suppression of ferroptotic stress or restoration of redox balance preferentially skews macrophages toward an M2 phenotype (Dong et al., 2026; Li H. et al., 2022; Yang et al., 2022; Chen et al., 2025). The Nrf2 pathway serves as a central node, upregulating antioxidant defenses (SLC7A11, GPX4, HO-1) to curb lipid peroxidation while antagonizing NF-κB-driven inflammation and promoting IL-10/TGF-β expression (Dong et al., 2020; Gao et al., 2021; Wang and He, 2022; Li J. et al., 2022). Similarly, PPARγ activation enhances cholesterol efflux and suppresses ACSL4-mediated peroxidation, thereby reinforcing M2 commitment and ferroptosis resistance (Chawla et al., 2001; Chen P. et al., 2023). Conversely, plaque hypoxia and metabolic stress activate HIF-1α, which synergizes with iron/ROS to fuel glycolytic reprogramming and M1 skewing; in contrast, IL-4/IL-13–induced STAT6 signaling bolsters M2 differentiation and antioxidant capacity, buffering against ferroptotic vulnerability (Aarup et al., 2016; Tawak et al., 2015; Wang et al., 2017; Ma et al., 2022). Notably, unmitigated ferroptotic stress not only depletes macrophage populations but also destabilizes the M2 phenotype, driving a maladaptive shift toward inflammation that accelerates plaque progression in AS (Peled and Fisher, 2014). Collectively, targeting ferroptosis-associated iron and redox dysregulation represents a promising strategy to modulate macrophage plasticity and restore vascular homeostasis in AS.

### 3.3 Ferroptosis promotes vascular smooth muscle cell proliferation

**FIGURE 1:**
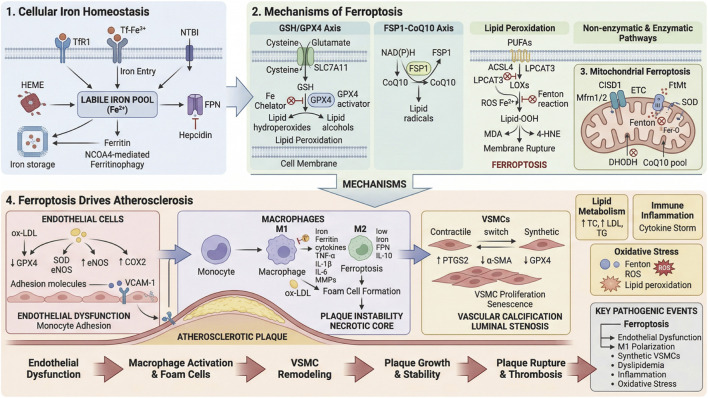
Ferroptosis promotes vascular smooth muscle cell proliferation.

Vascular smooth muscle cells (VSMCs) are located within the tunica media and play essential roles in regulating vascular tone and maintaining vascular elasticity. During the progression of multiple vascular diseases, VSMCs undergo a phenotypic switch from a contractile state to a synthetic state, accompanied by sustained proliferation and migration toward the intima (Bennett et al., 2016; Owens et al., 2004; Basatemur et al., 2019). At the same time, they release large amounts of extracellular matrix components, including elastic fibers, collagen, and MMPs, as well as cytokines such as monocyte chemoattractant protein-1 (MCP-1), IL-1β, and IL-6, thereby promoting vascular stiffening and calcification. Iron deposition, treatment with the ferroptosis inducers erastin and RSL3, or knockdown of the iron-regulatory protein SLC7A11 all accelerate this process (Kawada et al., 2018; Ye et al., 2022). Ferroptosis in VSMCs increases intracellular ROS accumulation, markedly upregulates the PTGS2, downregulates α-SMA and GPX4, and drives the phenotypic transition of VSMCs from the contractile to the synthetic state, thereby aggravating neointimal formation (Yu et al., 2025). Oxidative stress induces robust expression of HIF-1α, which can regulate HO-1 expression by binding to the promoter region of HO-1, thereby indirectly promoting heme degradation and the release of free iron, ultimately triggering ferroptosis in VSMCs (Song et al., 2023). Activation of ferroptotic signaling in VSMCs can also induce NAD^+^ depletion and the release of pro-inflammatory senescence-associated secretory factors (Sun et al., 2024). Intervention with the ferroptosis inhibitor liproxstatin-1 or forced overexpression of GPX4 can suppress NCOA4-mediated ferritinophagy through the PPARγ signaling pathway, thereby lowering intracellular iron levels, inhibiting the expression of senescence markers such as γH2A.X, p53, p21^WAF1^, p16^INK4A^, as well as NAD^+^-consuming proteins including PARP-1 and CD38, and consequently alleviating VSMC senescence and vascular stiffness (Sun et al., 2024; Ajoolabady et al., 2025; Yang Y. et al., 2026). Taken together, excessive iron promotes abnormal VSMC proliferation and vascular calcification, ultimately resulting in luminal stenosis and accelerating the progression of atherosclerotic plaques (Figure 1).

### 3.4 Ferroptosis regulates lipid metabolism

Dyslipidemia occupies a central role in AS, and excessive lipid deposition within the injured intima represents the earliest pathological event in atherogenesis. Ferroptosis driven by lipid peroxidation is closely linked to lipid dysregulation (Gaggini et al., 2022; Xu et al., 2023). Inhibition of ferroptosis has been shown to modestly reduce total cholesterol (TC) levels in ApoE^−/−^ mice, increase high-density lipoprotein cholesterol (HDL-C), and, importantly, significantly lower serum low-density lipoprotein cholesterol (LDL-C) and triglyceride (TG) levels (Jiang X. et al., 2024). Moreover, ferroptosis inhibitor ferrostatin-1 (Fer-1) enhances resistance to lipid peroxidation in AS mice through the Nrf2/FSP1 pathway, thereby significantly ameliorating the elevated plasma TG, TC, and LDL levels induced by a high-fat diet in the ApoE^−/−^ model (Wan et al., 2023; Bai et al., 2020; Emmanuel et al., 2022). At the immune-inflammatory level, ox-LDL activates TLR4/NF-κB signaling, promoting the transcription of pro-inflammatory cytokines such as IL-1β, IL-6, and TNF-α, which in turn exacerbate oxidative stress and iron dysregulation (Yang et al., 2014; Meng et al., 2013; Bhaskar et al., 2011; Zhang et al., 2021; Ouyang et al., 2021). Lipid peroxidation products such as MDA and 4-HNE can function as DAMPs, triggering NLRP3 inflammasome activation and further amplifying inflammatory cascades (Yeon et al., 2017; Uchida, 2013; Zhang J. et al., 2024). Notably, PPARγ activation has been shown to inhibit NCOA4-mediated ferritinophagy, thereby reducing intracellular iron availability and attenuating ferroptosis (Hu et al., 2025; Huang et al., 2025). In addition, PPAR signaling suppresses NF-κB–mediated inflammatory responses, leading to reduced secretion of IL-1β, IL-6, and TNF-α (Tovar et al., 2021; Ju et al., 2019). These findings suggest that PPARs integrate lipid metabolism, iron homeostasis, and immune regulation, thereby serving as key modulators of ferroptosis in atherosclerosis (Supplementary Table S1).

### 3.5 Ferroptosis amplifies immune inflammation

Inflammation plays a pivotal role in the initiation and progression of atherosclerosis, and ferroptosis is intimately intertwined with inflammatory processes. Lipid peroxidation and redox disequilibrium induced by ferroptosis can activate multiple inflammatory cell populations and signaling pathways; conversely, aberrant expression of pro-inflammatory mediators can influence intracellular iron levels by regulating ferritin synthesis (Chen Y. et al., 2023). In AS, increased levels of IL-6, IL-1β, TNF-α, and MDA, together with elevated Fe^2+^/Fe^3+^ and reduced GSH, reflect a state of ferroptosis-driven inflammatory imbalance (Gao et al., 2023). *In vitro*, ferric ammonium citrate (FAC) enhances lipid ROS accumulation and suppresses GPX4 in THP-1 macrophages, thereby promoting release of IL-1β and IL-18, consistent with inflammasome activation (Su et al., 2021). Furthermore, iron loading potentiates ox-LDL–induced macrophage activation via the TLR4/NF-κB pathway, linking iron metabolism to inflammatory signaling and foam-cell formation (Xiao et al., 2020). Conversely, inhibition of ferroptosis restores redox balance and suppresses inflammatory signaling. Ferroptosis inhibitors reduce NF-κB activation, decrease cytokine production (IL-1β, IL-6, TNF-α), and attenuate MMP-mediated extracellular matrix degradation (Sampilvanjil et al., 2020). Additionally, activation of the NRF2 pathway enhances antioxidant defenses (GPX4, SLC7A11), thereby suppressing both ferroptosis and inflammation. Environmental stressors further exacerbate this process (Song and Long, 2020; Li et al., 2025). For example, CdTe quantum dots induce ferroptosis through ERK1/2 activation and NRF2 inhibition, leading to ferritin degradation (FTH1), iron release, and intensified inflammatory responses (Liu et al., 2022).

### 3.6 Ferroptosis induces oxidative stress

Redox homeostasis is critical for physiological immune regulation, but excessive reactive oxygen species (ROS) drive macromolecular damage and cell death (Liu S. et al., 2023; Shields et al., 2021). In AS, oxidative stress is a pervasive driver that oxidizes LDL, impairs endothelial function, and accelerates foam cell formation and fibrous cap rupture (Munno et al., 2024; Marchio et al., 2019; Ajoolabady et al., 2024). Ferroptosis both arises from and exacerbates this oxidative milieu. Excess labile iron in AS lesions catalyzes ROS generation, elevating lipid peroxidation products (MDA, 4-HNE) while depleting GSH and SOD, thereby disrupting macrophage redox balance and accelerating plaque progression (Xu X. et al., 2024; Luo et al., 2024). Concurrently, GPX4 downregulation permits phospholipid hydroperoxide accumulation, directly linking ferroptotic susceptibility to endothelial dysfunction and foam cell formation (Coornaert et al., 2024). Mechanistically, ferroptosis aggravates AS-associated oxidative stress and lipid dyshomeostasis in ApoE^−/−^ mice via the TFR1/SLC11A2/GPX4 axis (Cao Y. et al., 2025). Conversely, ferroptosis inhibition restores GPX4/SOD activity, scavenges ROS, and suppresses plaque development (Jiang X. et al., 2024). Agents like quercetin and YAP1 further stabilize the GSH pool by upregulating GCLC/GCLM, protecting VSMCs from oxLDL-induced ferroptosis and enhancing plaque stability (Chen et al., 2024; Zhang J. et al., 2023). Collectively, the ferroptosis–oxidative stress axis forms a self-reinforcing loop that propagates cellular injury across all major AS lesion components, positioning redox-targeted ferroptosis modulation as a promising therapeutic strategy.

## 4 Conclusion

Ferroptosis has emerged as a mechanistically important driver of atherosclerosis, linking iron overload, lipid peroxidation, oxidative stress, and immune inflammation into a unified pathogenic framework. Rather than representing an isolated mode of cell death, ferroptosis intersects with multiple hallmarks of atherogenesis, including endothelial dysfunction, macrophage polarization, vascular smooth muscle cell phenotypic remodeling, lipid metabolic disturbance, and plaque destabilization. The current evidence indicates that excessive intracellular iron, impaired antioxidant systems such as GSH/GPX4, and dysregulated mitochondrial redox homeostasis jointly amplify ferroptotic injury within the vascular wall. Through these mechanisms, ferroptosis may not only accelerate lesion initiation and growth, but also promote necrotic core expansion, fibrous cap vulnerability, and thrombotic risk. These findings support the view that ferroptosis is not merely a bystander phenomenon in AS, but an active participant in disease progression.

From a therapeutic perspective, targeted inhibition of ferroptosis or restoration of redox–iron homeostasis offers a promising strategy to mitigate oxidative damage, suppress inflammatory amplification, and stabilize vulnerable plaques. Priority targets include the SLC7A11–GPX4 axis, NCOA4-mediated ferritinophagy, ACSL4-dependent lipid peroxidation, and mitophagy–ferroptosis crosstalk. Preclinical efficacy of natural compounds (quercetin) and small-molecule inhibitors (ferrostatin-1, liproxstatin-1) underscores the translational potential of ferroptosis modulation in AS. Future efforts should focus on cell-type-specific delivery, biomarker-guided patient stratification, and rational combination with established lipid-lowering or anti-inflammatory regimens. Ultimately, elucidating the spatiotemporal dynamics of ferroptosis within human atherosclerotic lesions, coupled with rigorous safety assessment, will be pivotal to harnessing this pathway for precision cardiovascular therapeutics.
